# The British Journals, &c.: Anatomy and Physiology

**Published:** 1842-04

**Authors:** 


### II. THE BRITISH JOURNALS, &c.

### ANATOMY AND PHYSIOLOGY.

Case of Paralysis of the Portia Dura, illustrative of a curious physiological fact. J. Ward, a marine, aged 33, came to the poorhouse with secondary syphilis, for which the ordinary treatment, consisting of an alterative course with sarsaparilla, was successfully used. But upon the subsidence of the syphilitic symptoms he was suddenly seized with a paralysis of some of the muscles of the face. His mouth was drawn to the left side, the natural power of winking his right eye was lost, and he could not elevate the right angle of his mouth. As the senses of smelling, sight, hearing, the motions of the eyeball, the motions and taste of the tongue, and the motion of the lower jaw were perfect on both sides, the paralysis was evidently confined to the portia dura of the right side.

But the most curious part of the case, and that for which I have been induced to relate it, is, that he could close the eyelids at will, while the involuntary winking was confined to the left eye, showing that the voluntary power of closing the eyelids is communicated by means of a different nerve from that which causes the natural winking, the latter depending upon the portia dura, while the former is probably derived from the ophthalmic nerve.—Dr. Zabriskie, *American Journal of the Medical Sciences,* No. iv. *Oct.* 1841.

Minute Anatomy of Fatty Degeneration of the Liver. The author observes that in order to make the subsequent description intelligible, he will premise a few words on the minute structure of the lobules of the liver.

Mr. Kiernan has well described the *vascular element* of these minute representatives of the organ. It consists of a capillary plexus intervening between the portal and hepatic veins. The diameter of the capillaries in this plexus is very large, being nearly twice that of a blood-globule; while the diameter of the capillaries in most other textures is the same as that of the blood-globule, and in some (as muscle) even less, so that the blood-globules only pass along by undergoing elongation. This large size of the capillaries of the liver, probably, has reference to the deficiency of propelling power in the portal circulation. This *portal hepatic plexus* may be termed *solid,* as it is extended in all directions, and presents areolæ of nearly the same dimensions in whatever plane it is cut. These areolæ are in general not larger than the diameter of the vessels which form them, so that a well-injected specimen might appear to be composed of little else than vessels.

In the interstices of this capillary plexus lies the *secreting portion of the bile-ducts.* If a thin section of an uninjected lobule be examined with a sufficient magnifying power, it is seen to be almost entirely made up of small, irregular, angular particles, each containing a circular or oval nucleus, within which is a minute point or two, the nucleolus. These particles have a determinate outline, are of some thickness, and possess a fine granular aspect. They also contain (which is very remarkable) one, two, or more globules of fatty matter, irregularly placed, and of somewhat variable bulk.

The microscope at once reveals the seat of the *fatty deposit in the diseased state of the organ.* Instead of containing a few minute scattered globules, *the nucleated particles are gorged with large masses of it,* which greatly augment their bulk, and more or less obscure their nuclei.

This simple description develops the whole anatomical condition of the disease, as well as explains its rougher characters, the bulk, the colour, and the freedom of the circulation. The particles, lying in the interstices of the capillary plexus, enlarge slowly and equably, in such a manner as to exert no injurious pressure on the vessels, while their new contents impart that peculiar hue which characterizes the disease. It also throws no little light on the nature and source of the disease. It seems to show that the fat is an *increase of a normal constituent,* and not a formation altogether unnatural in kind; thus distinguishing it from the fatty degenerations of other tissues, where fat is deposited in situations from which it is naturally absent. It likewise indicates an increased activity in the secreting action of the liver, for a considerable period before death, though why the *accumulation* of fat should occur within the nucleated particles does not so clearly appear. To explain that fully, will require a more complete knowledge than we yet possess, of the chemical affinities at play within these small laboratories of nature.—Mr. Bowman, *Lancet, Jan.* 1842.

---

On Fibre. The author observes that, in the mature blood-corpuscle, there is often seen a flat filament, already formed within the corpuscle. In mammalia, including man, this filament is frequently annular; sometimes the ring is divided at a certain part, and sometimes one extremity overlaps the other. This is still more the case in birds, amphibia, and fishes, in which the filament is of such length as to constitute a coil. This filament is formed of the discs contained within the blood-corpuscle. In mammals, the discs entering into its formation are so few as to form a single ring; and hence the biconcave form of the corpuscle in this class, and the frequent annular form of the filament it produces. In the other vertebrata, the discs contained within the blood-corpuscle are too numerous for a single ring; and they consequently form a coil. At the outer part of this coil, the filament, already stated to be flat, often presents its edge; whence there arises a greater thickness of the corpuscle, and an appearance of being cut off abruptly at this part; while in the centre there is generally found the unappropriated portion of a nucleus; and hence the central eminence, surrounded by a depression, in those corpuscles which, from the above-mentioned cause, have the edge thickened. The nucleus of the blood-corpuscle in some instances resembles a ball of twine, being actually composed, at its outer part, of a coiled filament. In such of the invertebrata as the author has examined, the blood-corpuscle is likewise seen passing into a coil.

The filament thus formed within the blood-corpuscle has a remarkable structure; for it is not only flat, but deeply grooved on both surfaces, and consequently thinner in the middle than at the edges, which are rounded; so that the filament, when seen edgewise, appears at first sight to consist of segments. The line separating the apparent segments from one another is, however, not directly transverse, but oblique.

Portions of the clot in blood sometimes consist of filaments having a structure identical with that of the filament formed within the blood-corpuscle. The ring formed in the blood-corpuscle of man, and the coil formed in that of birds and reptiles, have been seen by the author unwinding themselves into the straight and often parallel filaments of the clot; changes which may be also seen occurring in blood placed under the microscope before its coagulation; and similar coils may be perceived scattered over the field of view, the coils here also appearing to be altered blood-corpuscles, in the act of unwinding themselves; filaments, having the same structure as the foregoing, are to be met with apparently in every tissue of the body. The author enumerates a great variety of organs in which he has observed the same kind of filaments.

Among vegetable structures, he subjected to microscopic examination the root, stem, leaf-stalk, and leaf, besides the several parts of the flower; and in no instance of phanerogamous plants, where a fibrous tissue exists, did he fail to find filaments of the same kind. On subsequently examining portions indiscriminately taken from ferns, mosses, fungi, lichens, and several of the marine algæ, he met with an equally general distribution of the same kind or filaments. The flat filament seen by the author in all these structures, of both animals and plants, he states to be that usually denominated a fibre. Its appearance is precisely such as that of the filament formed within the corpuscle of the blood. It is known, he remarks, that discoid corpuscles circulate in plants; and it remains to be seen whether or not filaments are formed also in these.

By gradually tracing the fibre or filament above mentioned into similar objects of larger size, the author endeavours to show that it is not possible to draw a line of separation between the minutest filament, and an object being to all appearance composed of two spirals running in opposite directions, and interlacing at certain regular intervals; an arrangement which produces in the entire object a flattened form, and gives it a grooved appearance. It is, in fact, the structure which, for want of a better term, he has called a flat filament. The edge of this filament presents what, at first sight, seem like segments, but which, in reality, are the consecutive curves of a spiral thread. A transverse section of such an object is rudely represented by the figure 8. This is also precisely the appearance presented by the minutest filament, generally termed fibre: and the author particularly refers to the oblique direction of the line separating the apparent segments in the smaller filament, in connexion with the oblique direction of the spaces between the curves of the spiral threads in the larger one.

The spiral form, which has heretofore seemed wanting, or nearly so, in animal tissues, is then shown to be as general in animals as in plants. Nervous tissue, muscle, minute blood-vessels, and the crystalline lens, afford instances in proof of this. And if the author's view of identity in structure between the larger and the smaller filaments be correct, it follows that spirals are much more general in plants themselves than has been hitherto supposed; spirals would thus appear, in fact, to be as universal as a fibrous structure.

The tendency to the spiral form manifests itself very early. Of this the most important instance is afforded by the corpuscle of the blood, as above described. The author has also obtained an interesting proof of it in cartilage from the ear of a rabbit; where the nucleus, lying loose in its cell, resembled a ball of twine, being composed at its outer part of a coiled filament, which it was giving off to weave the cell-wall; this cell-wall being no other than the last-formed portion of what is termed the intercellular substance—the essential part of cartilage. These nuclei in cartilage, as well as those in other tissues, there is ground for believing to be descended, by fissiparous generation, from the nuclei of blood-corpuscles.

The author then describes the mode of origin of the flat filament or fibre, and its reproduction in various animal and vegetable tissues, which he enumerates. He conceives that each filament is a compound body which enlarges, and, from analogy, may contain the elements of future structures, formed by division and subdivision, to which no limits can be assigned.

He then traces the formation of muscle out of cells, which, according to his observations, are derived from corpuscles of the blood, to the state where there exists what is denominated the fibril. In this process there are to be observed the formation of a second order of tubes within the original tube; a peculiarly regular arrangement of discs within these second tubes; the formation, first of rings and then of spirals, out of discs so arranged; the interlacing of the spirals; and the origin, in the space circumscribed by these, of spirals having a minuter size; which in their turn surround others still more minute; and so on. The outer spirals enter for the most part into the formation of the investing membrane discovered by Schwann, but for the only complete description of which, in a formed state, we are indebted to Mr. Bowman. The inner spirals constitute what are denominated the fibrillæ. The fibril appears to the author to be no other than a state of the object which he designates a flat filament; and which, as he shows, is a compound structure. The fibril he finds to be, not round and beaded, as it has been supposed, but a flat and grooved filament; the description above given of the structure of the filament being especially applicable here. This flat filament is so situated in the fasciculus of voluntary muscle, as to present its edge to the observer. It seems to have been the appearance presented by the edge of this filament, that is to say, by the curves of a spiral thread, that suggested the idea of longitudinal bead like enlargements of the fibril, as producing striæ in the fasciculus of voluntary muscle. In the author's opinion, the dark longitudinal striæ are spaces (probably occupied by a lubricating fluid) between the edges of flat filaments, each filament being composed of two spiral threads, and the dark transverse striæ rows of spaces between the curves of these spiral threads. The filament now mentioned, or its edge, seems to correspond to the primitive marked thread or cylinder of Fontana—to the primitive fibre of Valentin and Schwann—to the marked filament of Skey—to the elementary fibre of Mandl—to the beaded fibril of Schwann, Müller, Lauth, and Bowman—and to the granular fibre of Gerber. The changes known to be produced by the alternate shortening and lengthening of a single spiral are exhibited in the microscope by a fasciculus of spirals, not only in its length and thickness, but in the width of the spaces (striæ) between the curves of the spirals. And a muscle being no other than a vast bundle of spirals, it is in contraction short and thick; while in relaxation it is long and thin; and thus there occurs no flattening of bead-like segments in contraction. The author has found no segments that could undergo this change. These observations on the form of the ultimate threads in voluntary muscle were first made on the larva of a Batrachian reptile; and have been confirmed by an examination of this structure in each class of vertebrated animals, as well as in the crustacea, mollusca, annelida, and insects.

He finds that the toothed fibre, discovered by Sir David Brewster in the crystalline lens, is formed out of an enlarged filament; the projecting portions of the spiral threads in the filament, that is, the apparent segments, becoming the teeth of that fibre.

The compound filaments are seen with peculiar distinctness in the blood-vessels of the arachnoid membrane. In connexion with the spiral direction of the outer filament in these vessels, the author refers to the rouleaux in which the red blood-discs are seen to arrange themselves, in the microscope, as probably indicating a tendency to produce spiral filaments. To form rouleaux, corpuscle joins itself to corpuscle, that is to say, ring to ring; and rings pass into coils. The union of such coils, end to end, would form a spiral. But the formation by the blood corpuscles of these rouleaux is interesting in connexion with some facts recorded by the author in a former memoir; namely, that many structures, including blood-vessels, have their origin in rows of cells derived from corpuscles of the blood. The human spermatozoon presented a disc with a pellucid depression, each of the two sides of the peripheral portion of which was extended into a thread; these two threads forming by being twisted the part usually designated as the tail. The occurrence of two tails, observed by Wagner, is accounted for by the author by the untwisting of these threads.

The author has noted very curious resemblances in mould, arising from the decay of organic matter, to early stages in the formation of the most elaborate animal tissues, more particularly nerve and muscle. Flax has afforded satisfactory evidence of identity, not only in structure, but in the mode of reproduction, between animal and vegetable fibre.

Valentin had previously stated that in plants all secondary deposits take place in spiral lines. In the internal structure of animals, spirals have heretofore seemed to be wanting, or very nearly so. Should the facts recorded in this memoir, however, be established by the researches of other investigators, the author thinks the question in future may perhaps be, where is the "secondary deposit" in animal structure, which is not connected with the spiral form? The spiral in animals, as he conceives he has shown, is in strictness not a secondary formation, but the most primary of all; and the question now is whether it is not precisely so in plants.

In a postscript the author observes, that there are states of voluntary muscle in which the longitudinal filaments ("fibrillæ") have no concern in the production of the transverse striæ; these striæ being occasioned by the windings of spirals, within which very minute bundles of longitudinal filaments are contained and have their origin. The spirals are interlaced. When mature, they are flat and grooved filaments, having the compound structure above described. With the shortening of the longitudinal filaments ("fibrillæ") in muscular contraction, the surrounding spirals, and of course the striæ, become elongated and narrow; while in relaxation these changes are reversed.—Dr. BARRY'S *Paper, Proceedings of the Royal Society,* No. li. 1841-2.

[In consequence of the very great importance of the subject treated of in this paper, we give it in full.]

Asymmetrical Development. There was presented to the American Philosophical Society a lower jaw (fossil) belonging to the genus Tetracaulodon. This is remarkable for having a single alveolus for a tusk ; this alveolus is on the right side, and there is not the slightest trace of a corresponding alveolus ever having existed on the other.—*Proceedings of the American Philosophical Society*, No. xix. *Oct.* 1841.

### PATHOLOGY, PRACTICAL MEDICINE, AND THERAPEUTICS.

